# Bilateral lower limb edema induced by rapid improvement of glycemic control with insulin therapy in a subject with poorly controlled type 2 diabetes

**DOI:** 10.1007/s00592-017-0997-7

**Published:** 2017-04-27

**Authors:** Yuki Kan, Takatoshi Anno, Fumiko Kawasaki, Maiko Takai, Ryo Shigemoto, Hideaki Kaneto, Kohei Kaku, Niro Okimoto

**Affiliations:** 1 Department of General Internal Medicine 1, Kawasaki Medical School, 2-6-1 Nakasange, Kita-ku, Okayama, 700-8505 Japan; 2 Department of Diabetes, Metabolism and Endocrinology, Kawasaki Medical School, Kurashiki, 701-0192 Japan

Dear Editor,

A 57-year-old man who had 5-year history of type 2 diabetes (T2DM) but hesitated to receive its treatment was referred to our department. His height and body weight were 164.0 cm and 48.0 kg. His vital signs were: heart rate 75 beats/min, blood pressure 122/65 mmHg, and temperature 36.9 °C. Non-fasting plasma glucose was markedly elevated to 579 mg/dL. HbA1c and glycoalbumin levels were also markedly elevated to 19.4 and 110.1%. Serum insulin level was as low as <1.0 μU/mL. β-Hydroxybutyrate and acetoacetate were 267.9 and 172.6 μmol/L. Anti-GAD antibody was negative. Renal and liver function was within normal range. Brain natriuretic peptide (BNP) was 63.5 pg/mL. Urinary albumin excretion was 234 mg/g creatinine, and urine protein (3+) was detected in urinalysis.

**Fig. 1:**
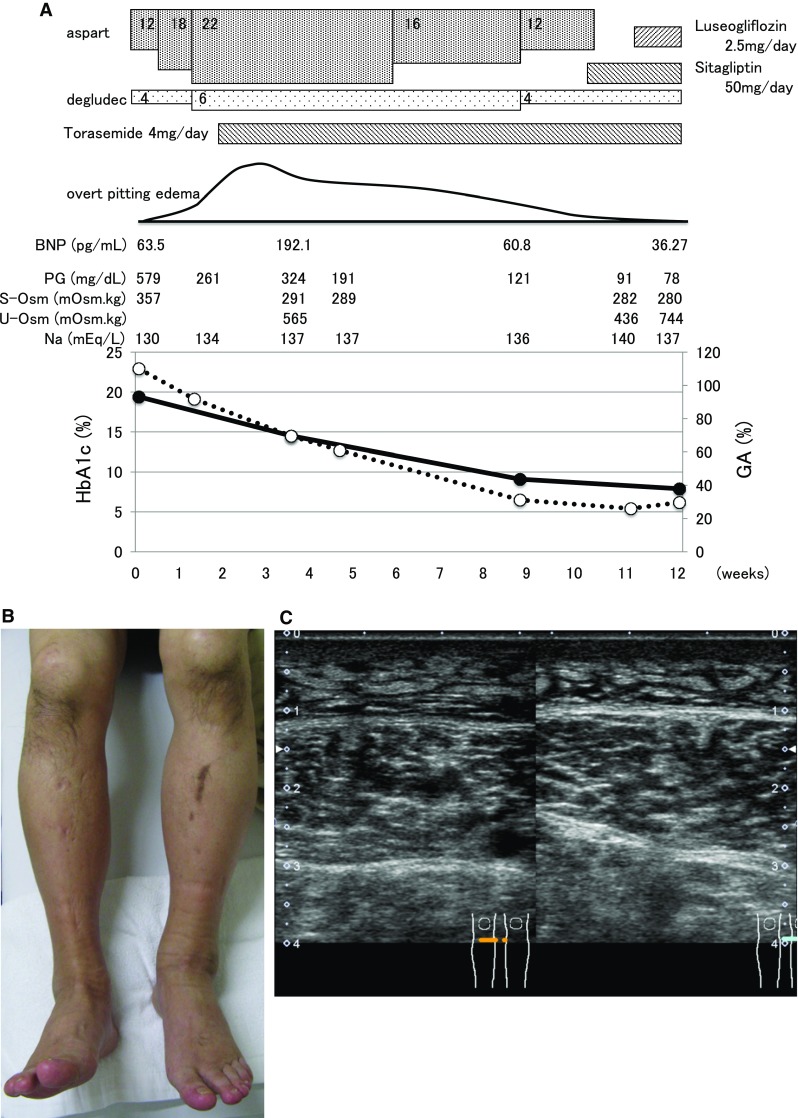
**a** Time course of various clinical parameters in this subject. **b** Bilateral edema in ankles and pretibial region. **c** Lower limb ultrasound revealing fluid accumulation under subcutaneous fat in the bilateral lower limbs

His life style was very poor; for example, he drunk 1–2 L of PET bottle of juice and ate a lot of snacks and fruits. At first, we treated him with diet therapy of 1600 kcal/day (about 28 kcal/ideal body weight kg) and sodium restriction of 6 g/day. In addition, we started basal insulin (4 units of degludec) once a day and bolus insulin (4 units of aspart) before each meal. He was followed up as an outpatient, and we increased insulin dose gradually each 1 week. And we treated him with 6 units of degludec and 22 units of aspart (8, 6, and 8 units before breakfast, lunch, and dinner) for 2 weeks. HbA1c and glycoalbumin levels were very rapidly decreased only in 3 weeks from 19.4 to 14.5% and from 110.1 to 69.5%, respectively (Fig. 1a). After starting insulin therapy, overt pitting edema was observed in both legs (Fig. 1b). Lower limb ultrasound clearly revealed the fluid accumulation under subcutaneous fat in the bilateral lower limbs (Fig. 1c). In addition, his body weight was increased by 7 kg only for 2 weeks. In an echocardiogram, his heart movement was normal and ejection fraction (EF) was 62.1%, although BNP level was increased to 192.1 pg/mL. Renal and liver function was within normal range. TSH and FT4 levels were 2.726 μIU/mL and 0.73 ng/dL. Urine protein (2+) was detected in urinalysis. Even after starting treatment with 4 mg/day of torasemide, his leg edema was not reduced. Since his glycemic control was improved, we reduced insulin to 4 units of degludec and 16 units of aspart. Without changing to another kind of insulin, his bilateral lower limb edema was markedly improved and BNP level was decreased to 60.8 pg/mL.

It is known that insulin therapy increases the reabsorption of sodium at the level of the renal tubule which leads to the development of insulin edema. In addition, it is known that insulin treatment leads to hyperaldosteronism which could be also involved in the development of insulin edema. There were a few reports about insulin edema all of which were reduced after changing to another kind of insulin [1–4]. In this case, however, bilateral lower limb edema was markedly improved without changing to another kind of insulin. Therefore, we think that bilateral lower limb edema in this subject was induced by the rapid improvement of glycemic control rather than the introduction of insulin therapy *per se*. There are several possible mechanisms for the development of edema induced by rapid improvement of glycemic control. For example, it is possible that rapid improvement of glycemic control reduces plasma osmolality which leads to intracellular water retention and brings out edema. Indeed, plasma osmolality in this subject was decreased after the improvement of glycemic control (Fig. 1a). It remains unknown, however, why such rare phenomena were observed in this subject. In addition, it is possible that the difference in salt sensitivity is, at least in part, involved in the development of such phenomena, but it remains unknown whether such phenomena are relatively easily induced in Japanese subjects whose salt sensitivity is thought to be different from those in Caucasians. In order to address this point, it would be necessary to perform future clinical study with large population. Taken together, we assume that the edema in this subject was induced by the alteration of various parameters after rapid improvement of glycemic control such as plasma osmolality rather than by the introduction of insulin therapy per se.

In conclusion, we should keep in mind the possibility that when we try to obtain good glycemic control with insulin therapy especially in subjects with poorly controlled diabetes, edema is induced not only by the introduction of insulin therapy per se but also by the alteration of various parameters after rapid improvement of glycemic control such as the alteration of plasma osmolality.
